# Editorial: Specific targeting of MHC antigens for T-cells and immune cells in human disease

**DOI:** 10.3389/fimmu.2024.1540449

**Published:** 2025-01-06

**Authors:** Ali Afzal, Muhammad Babar Khawar, Weijuan Gong, Brian M. Baker, Haibo Sun

**Affiliations:** ^1^ Institute of Translational Medicine, Medical College, Yangzhou University, Yangzhou, China; ^2^ Jiangsu Key Laboratory of Experimental & Translational Non-Coding RNA Research Yangzhou, Yangzhou, China; ^3^ Applied Molecular Biology and Biomedicine Lab, Department of Zoology, University of Narowal, Narowal, Pakistan; ^4^ Department of Health Management Center, Affiliated Hospital of Yangzhou University, Yangzhou University, Yangzhou, Jiangsu, China; ^5^ Department of Basic Medicine, Medical College of Yangzhou University, Yangzhou University, Yangzhou, Jiangsu, China; ^6^ Yangzhou Key Laboratory of Pancreatic Disease, Institute of Digestive Diseases, Affiliated Hospital of Yangzhou University, Yangzhou University, Yangzhou, Jiangsu, China; ^7^ Department of Chemistry and Biochemistry, University of Notre Dame, Notre Dame, IN, United States; ^8^ Harper Cancer Research Institute, University of Notre Dame, Notre Dame, IN, United States

**Keywords:** major histocompatibility complex, MHC-I, MHC-II, immune system, T-cells

Our immune system is a complex network that enables an organism to differentiate between “self” and “non-self”. This function maintains homeostasis and protects the body from pathogenic threats. While the systems in humans and other vertebrate animals are complex and multi-armed, even ancient organisms such as sea sponges, possess rudimentary immune systems. The capacity to differentiate between self and non-self is foundational to the viability of more complex multicellular life (1).

**Figure 1:**
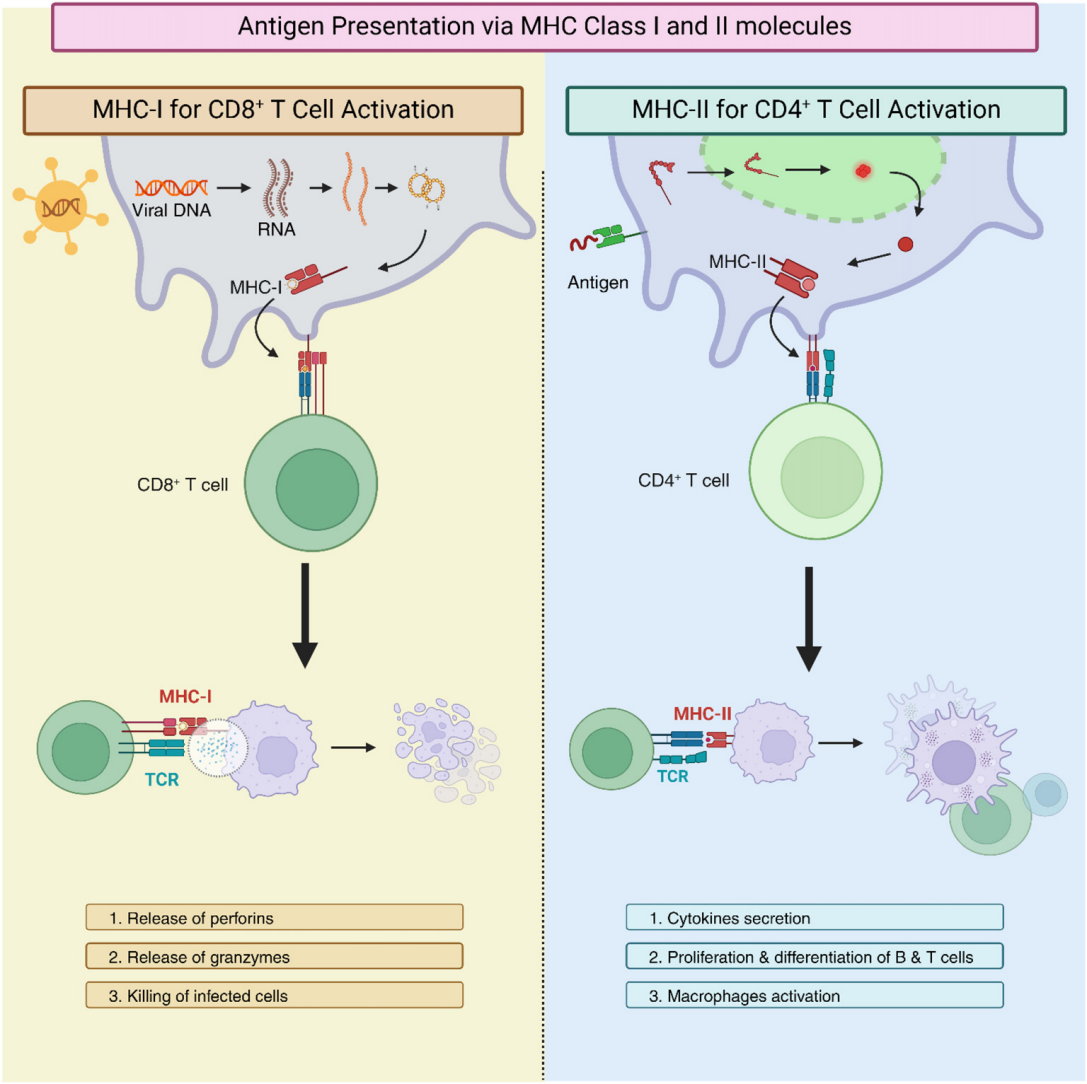
Mechanisms of antigen presentation via major histocompatibility complex class I (MHC-I) and class II (MHC-II). The left panel illustrates MHC-I presentation, where intracellular proteins are processed into peptides that bind to MHC-I molecules and are presented on the cell surface for recognition by CD8+ cytotoxic T lymphocytes. The right column depicts MHC-II presentation, occurring on specialized antigen-presenting cells (APCs), where extracellular antigens are processed and displayed on MHC-II molecules for recognition by CD4+ helper T lymphocytes. In both pathways, T cell receptors (TCRs) engage with the peptide-MHC complexes and initiate immune signaling and T cell activation.

In vertebrate animals, antigen presentation via the highly polymorphic major histocompatibility complex class I (MHC-I) and class II (MHC-II) proteins is a key component of adaptive immunity (2). These proteins display peptide fragments on the cell membrane so that T cells may recognize them to initiate an immune response. For MHC-I, which is found on all nucleated cells, intracellular proteins act as sources of these peptides, allowing CD8^+^ cytotoxic T lymphocytes to identify and eliminate infected or malignant cells (3). On the other hand, MHC-II proteins are expressed on specialized antigen-presenting cells (APCs), such as B lymphocytes, dendritic cells (DCs), and macrophages, and present peptides from exogenous proteins, allowing CD4^+^ helper T lymphocytes to coordinate broader immune responses (4). For both CD8^+^ and CD4^+^ T cells, T cell receptors (TCRs) bind to specific peptide-MHC complexes, triggering a signaling cascade and leading to T cell activation and subsequent downstream responses (**Figure 1**).

Dysregulation of MHC function is implicated in various human diseases (4). Autoimmune disorders, for instance, can result from genetic polymorphisms in MHC genes that cause erroneous targeting of self-tissues (5, 6). Altered MHC expression is also linked to the evasion of immune surveillance in viral infection and cancer which enables diseased cells to escape recognition and destruction by the immunocytes (7). Additionally, specific MHC alleles are linked to enhanced vulnerability to some viral infections, including HIV, and hepatitis B and C (8). The development of tailored immunological therapeutics for these conditions thus requires a thorough understanding of the molecular mechanisms driving T cell activation and MHC antigen presentation.

A promising path for therapeutic innovation in a variety of diseases is provided by research on MHC polymorphisms, their presented peptides, and how they are recognized by T cells. Our Research Topic, “*Specific Targeting of MHC Antigens for T-cells and Immune Cells in Human Disease*” delves into novel and precise methods of peptide-MHC targeting and modulating their recognition. We seek to contribute to the development of personalized therapies that enhance immune responses against viral infections, autoimmune disorders, and cancer by focusing on the molecular mechanisms governing peptide-MHC presentation, recognition, and T cell activation.

This Research Topic features five seminal papers that contribute to the understanding of immune modulation by investigating how enhancing TCR specificity via framework engineering can result in more precise and focused immune responses. It also examines exosomal membrane proteins for how they are composed, their physiological aspects, and therapeutic applications. Additionally, our Research Topic explores a key challenge in alloimmunity by explaining the use of IL-2 and TGF-β loaded nanoformulations to support transplantation tolerance. The implications of the MHC-restricted immunopeptidome in transplantation are also explored, particularly its role in transplant rejection and tolerance. Finally, the MHC-I/LILRB1 pathway is explored as a potential innate immune checkpoint in cancer which offers insights into cancer immunotherapy. Together, this Research Topic presents innovative approaches for modulating immune responses and advancing the treatment of human diseases.

In their research, Rosenberg et al. explored tuning TCR specificity by introducing mutations in regions far from the binding surface. Deep mutational scanning of the HIV-specific 868 TCR revealed ways to reduce recognition of SL9 escape variants without altering affinity towards the native epitope. Simulations suggest that this mutation restricts loop motions, limiting TCR binding to diverse ligands. This study offers a potential strategy to enhance TCR specificity for use in immunotherapy.

Xu et al. comprehensively reviewed the vital role of exosomes in numerous biological processes, including disease progression, immune responses and human development with their surface proteins being key contributors to these functions (10). These proteins facilitate various functions, for instance, communicating between cells, mediating recognition of target cells, and regulating immune responses. Notably, these surface proteins on cancer-derived exosomes have emerged as potential biomarkers for early cancer detection. This review delves into the composition and physiology of exosome-surface proteins and highlights their importance in various physiological and pathological contexts. By exploring these proteins, the authors aim to lay the groundwork for the development of novel diagnostic tools and therapeutic strategies in biomedicine.

Horwitz et al. have reported their groundbreaking findings in the form of a brief report in which they evaluated the potential of IL-2 and TGF-β loaded nanoformulations. These nanoformulations have previously shown to induce polyclonal T regulatory lymphocytes (Tregs) to help prevent allograft rejection and protect against graft-versus-host disease. They used a murine model of alloimmunization and demonstrated that the treatment with tolerogenic nanoformulations significantly inhibited the mixed lymphocyte reaction to alloantigens from donors without affecting responses to third-party antigens. This reduction in alloreactivity was accompanied by a 4 to 5-fold augmentation in CD4^+^ and CD8^+^ Tregs and a shift of recipient DCs toward a tolerogenic phenotype. Together these provide explanation of tolerogenic nanoformulations which can promote alloantigen tolerance by inducing Tregs as well as modulating DC function. This study provides a potential strategy to reduce allograft rejection and the need for long-term immune suppression.

Zhanzak et al. have critically assessed the emerging role of immunopeptidome in transplantation and focuses on its promise to target alloreactive T lymphocytes and improve transplant outcomes. The immunopeptidome, consisting of donor-peptides and MHC proteins is critical in immune surveillance and plays a critical role in mediating T lymphocyte responses in transplantation. MHC-derived antigens represent an important subset of peptides that can be crucial in triggering alloreactive responses. Gaining a more comprehensive understanding of the immunopeptidome in the context of transplantation could provide novel approaches for identifying, characterizing, and quantifying T lymphocytes that are specific to the donor. This exploration paves the way for personalized immunotherapies aimed at preventing rejection and promoting long-term allograft tolerance.

In a succinct mini-review, Hu et al. highlighted the role of immune checkpoint blockades (ICBs) as essential components in the treatment of advanced cancer, as they enhance anti-cancer adaptive immunity. However, their effectiveness is confined to a specific group of patients, and relapse frequently occurs. Recent discoveries have highlighted the role of ICBs in MHC-I/LILRB1 axis. Cancer cells with MHC-I interact with immunocytes with LILRB1 and deliver inhibitory signals to facilitate tumors escape immune surveillance. Their review explores the MHC-I/LILRB1 axis in cancer immune evasion and describes the therapeutic potential of blocking this interaction to improve cancer treatment outcomes. However, given the nascent stage of this field, further research is needed to assess its clinical efficacy, safety, and the potential for combining it with existing adaptive immune checkpoint therapies.

To sum up, this Research Topic highlights recent advancements in immune modulation and therapeutic strategies aimed at improving precision immunotherapy and the findings reported in this Research Topic represent a range of innovative approaches with the potential to enhance cancer treatment, transplant tolerance, and immune regulation. The continued integration of these innovative strategies promises to revolutionize the way we approach disease treatment, making therapies more effective, personalized, and less reliant on broad immune suppression. Looking ahead, the field of immune modulation holds tremendous promise for advancing personalized therapies across a range of diseases, including cancer, autoimmune disorders, and transplantation.
